# Invasive plasmids as ecosystem engineers—from mechanism to application

**DOI:** 10.1042/EBC20250040

**Published:** 2026-09-02

**Authors:** Solomon Garland, Victoria T. Orr, James P.J. Hall, Ellie Harrison

**Affiliations:** 1 School of Biosciences, The University of Sheffield, Sheffield, U.K.; 2 Institute of Infection, Veterinary and Ecological Sciences, University of Liverpool, Liverpool, U.K.

**Keywords:** bioaugmentation, community, horizontal gene transfer, microbiome, Plasmids

## Abstract

Horizontal gene transfer, mediated by mobile genetic elements such as conjugative plasmids, is recognised as a major driver of bacterial innovation. While predominantly explored in the context of change within individual strains and species, the broad host ranges of many plasmids mean that they can invade not just lineages but communities. This has far-reaching implications for both the fate of the plasmid and our understanding of bacterial adaptation, as well as applications for the functional engineering of microbial communities. In comparison to single-strain systems, in which plasmid invasion is largely determined by a now well-defined set of parameters—conjugation rate, fitness cost of carriage, and segregation loss—the spread of plasmids into communities is vastly more complex: governed by the wide range of dynamics within strains, but also by community dynamics, spatial heterogeneity, and the interactions between strain- and community-level selection. Here, we review the processes by which plasmids can invade communities and discuss how community complexity both constrains and facilitates plasmid spread. We further explore how this mechanistic understanding can be harnessed to enhance microbial community function.

## Introduction

**Figure 1:**
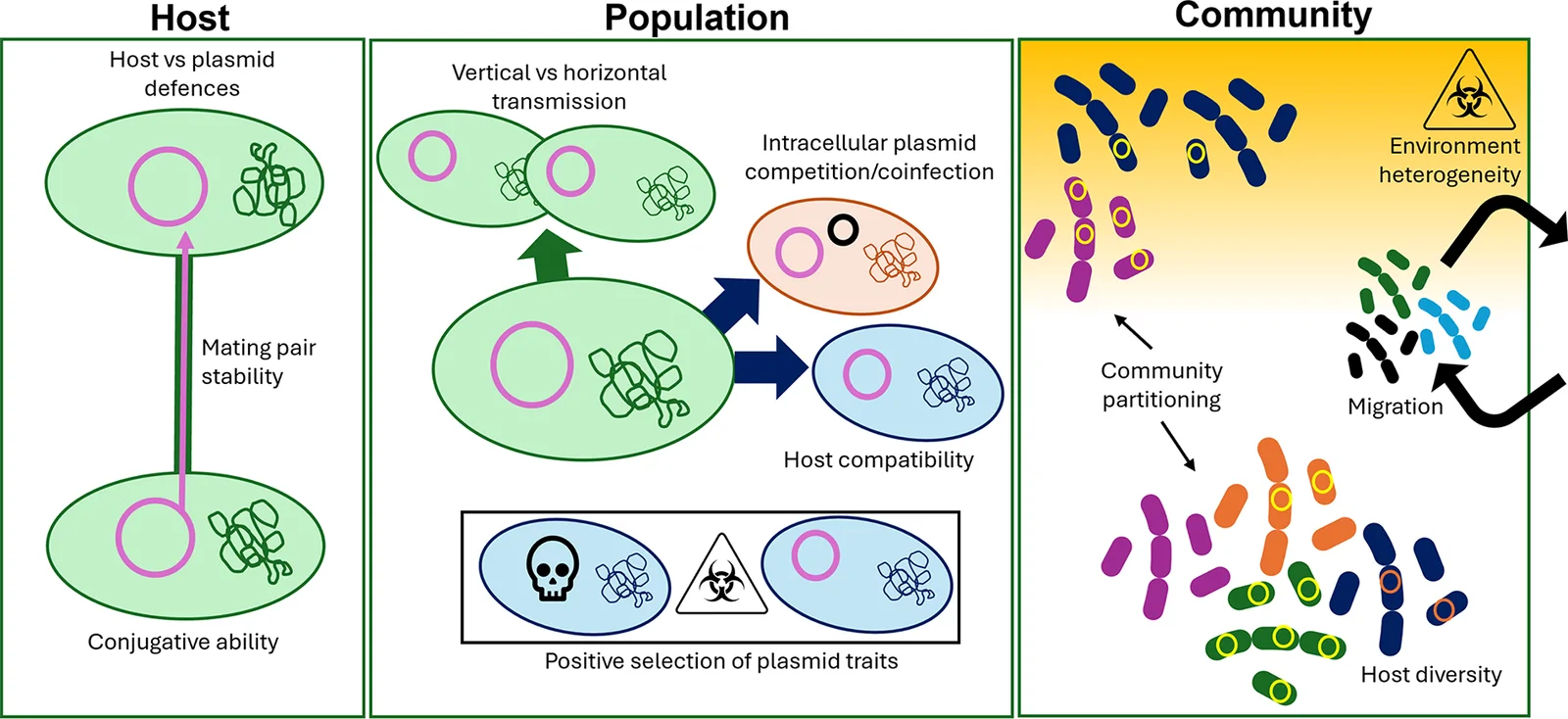
Conceptual overview Plasmid invasions can be considered on three levels: through invasion into a new host, invasion into a population, and invasion into a community.

**Conjugative plasmids** are ecosystem engineers, responsible for introducing many evolutionarily relevant traits [1] into bacterial **hosts**, **populations**, and **communities** via horizontal gene transfer (HGT) (Figure 1). Much of our understanding of plasmid dynamics has developed through experimental and theoretical studies of relatively simple single-population systems, through which the existence conditions for plasmid persistence have been defined. For example, the ‘plasmid paradox’, which describes the prediction that plasmids cannot be maintained in populations owing to their costs of carriage, can be solved by incorporating evolution into ecological frameworks [2,3]. However, single-species models overlook the capacity of plasmids to drive innovation in multi-species communities, and the challenges of plasmid persistence in a real-world context. Variation in plasmid attributes across species and strains, including host range, conjugation rates, fitness costs, and the marginal benefits of plasmid accessory genes [4–6] mean that community-level plasmid dynamics can differ markedly from those observed in single-strain systems. Beyond merely increased diversity, natural communities are shaped by interactions between strains that can shape and be shaped by plasmid spread [2,3,7]. To understand plasmid persistence and spread in nature, we must therefore look beyond population-level dynamics, to wider community-level processes.

Understanding the **invasion** of plasmids into a resident community is an important practical concern due to the threat posed by plasmid-mediated spread of antimicrobial resistance (AMR) [8–10]. However, plasmid invasion also offers opportunities to draw on the innovative power of conjugative plasmids to introduce valuable novel functions to a community. For example, plasmids might be enlisted to accelerate the degradation of pollutants [11–13], optimise crop-bacteria symbiosis [14], or replenish feedstocks from waste [15].

A plasmid can become established in a new community simply by virtue of its host being able to invade, which we term ‘**invasion-by-carriage**’. Here, plasmid invasion success would be completely aligned with that of its host, and can be understood within the framework of microbial invasions [16] (Box 1). However, HGT offers plasmids an alternative pathway for invasion, through infection of residents within the target microbiome, which we term ‘**invasion-by-transfer**’. Here, a plasmid may persist in a community long after its original host has been outcompeted [17]. Even where an original host can persist, a plasmid may enjoy greater success through horizontal transmission [18]. These processes are not mutually exclusive: a plasmid might initially transfer into a competitive strain before spreading by virtue of its host’s features, or might be carried into a community by a successful invader before spreading out to other potential hosts. As invasion-by-carriage can be largely understood through existing invasion frameworks, we focus here on invasion-by-transfer, which starts with the colonisation of a novel bacterial host.

Box 1 **Defining plasmid invasions**

*Useful definitions of invasion and related terms have been proposed by Kinnunen and colleagues* [16] *in a microbial context. Similar terms can be defined to cover invasion by plasmids and other mobile genetic elements*.
**Conjugative plasmids:** Extrachromosomal mobile genetic elements carrying genes for contact-dependent transmission between cells.
**Invasion:** Entry and establishment of an alien (i.e. previously not present in this environment) plasmid in a resident community.
**Establishment:** The persistence of the plasmid over long time periods in one or more community members.
**Host:** An individual bacterium at the point of plasmid entry: establishment within a host, for plasmids, is technically within a host lineage, i.e. daughter cells arising from the host.
**Permissive host:** A host which can stably maintain a plasmid over many generations.
**Population:** All members of a given strain in a habitat. Establishment in a population can involve vertical (outcompeting plasmid-free kin) and horizontal (infecting plasmid-free kin) transmission
**Community:** All members of all bacterial species in a habitat. Invasion into a community requires horizontal transmission between species.
**Community invasion:** Involve the stable maintenance of the plasmid within *multiple* members of the community (even if these hosts vary through time).
**Invasion-by-carriage:** The process by which a plasmid becomes established in a community by virtue of its being carried by a successfully invading host.
**Invasion-by-transfer:** The process by which a plasmid becomes established in a community by virtue of its transfer into one or more resident members of the community.

Here, we review the dynamics of plasmid invasion, from **establishment** within individuals and populations, scaling up to community-level interactions, and explore how this knowledge can be translated into rational strategies for community-level biotechnological intervention.

## Plasmid establishment within hosts

Plasmid invasions begin with plasmid transfer, which is primarily achieved through conjugation, thus requiring an encounter between a donor and a suitable recipient. The probability of such contact is strongly affected by the surrounding environment. Structured environments, such as biofilms, and/or areas of high microbial density, such as nutrient hot-spots, promote close, stable contact and efficient conjugation, though can limit encounters with new (plasmid-free) recipients. Unstructured environments, such as liquids, can increase population mixing but reduce cell–cell contact and local bacterial density, limiting spread [19,20]. Indeed, there is evidence that plasmids can themselves influence bacterial phenotypes such as biofilm production and motility by interfering with bacterial gene regulation, enhancing subsequent donor-recipient encounter probability [21].

Plasmid conjugation is costly for a donor [22]. Consequently, plasmids can employ quorum-sensing-based regulation of conjugation machinery, for example, to detect plasmid-free recipients [23,24] or environments associated with plasmid-encoded functions where transfer may be more advantageous [25]. Tight regulation restricts the burden of conjugation to only those conditions where transfer is likely to be successful.

Once initiated, mating-pair stabilisation—the stabilisation of initial contact between the donor conjugative pilus and the recipient cell—is influenced by compatibility between donor and recipient cell-surface traits. Disruption of this process can reduce conjugation efficiency, particularly in turbulent settings such as wastewater and intestinal lumen [26–28]. In the narrow host range F-type plasmids, for example, plasmid-encoded TraN variants, expressed on the donor cell-surface, define compatibility through interactions with the similarly variable Omp membrane pores of recipients [29]. Plasmids also encode entry and surface exclusion mechanisms that protect recipients from membrane disruption associated with conjugation attempts (‘lethal zygosis’) and, crucially, deny access to incompatible plasmids that could destabilise the occupant [30,31].

Successful DNA transfer does not always result in successful plasmid acquisition however. Genome defence systems—including restriction-modification and CRISPR—can degrade incoming DNA, including plasmids. In turn, many plasmids encode anti-defence systems such as methylases and anti-CRISPR proteins to overcome such barriers [32–34]. Once inside, a plasmid must interface with host machinery to replicate, and upon cell division, distribute between daughter cells to avoid segregational loss. Consequently, it has been observed that successful plasmid acquisition is more likely between related hosts [35,36]. Firstly, shared defence systems mean a plasmid capable of infecting one host is likely to infect closely related recipients [36]. Secondly, dependence on host factors for replication and expression [35], or adaptation to host genomes through the amelioration of plasmid–host conflicts [37] may also increase the probability of functional stability in related hosts.

Interactions with other mobile genetic elements already present in the cell can have both positive and negative effects on plasmid spread. Incompatible plasmids—sharing conjugation or replication machinery—can compete and destabilise each other within shared hosts [38]. In populations where carriage of a specific group of plasmids is common, e.g. IncF plasmids in human-associated *Enterobacterales* [39,40], plasmids of that group may struggle to invade. However, a recent study of intracellular competition between incompatible plasmids found that transient plasmid co-existence was possible [41]. Therefore, without a highly effective exclusion mechanism, incompatible plasmids could potentially displace a resident plasmid. Plasmids themselves also encode defence and antidefence systems to compete with each other [42]. Plasmid-borne defence systems such as CRISPR–Cas Type IV frequently target competitor plasmids; however, other plasmid features, such as toxin–antitoxin systems, penalise cells that lose resident plasmids [43,44], effectively blocking the invasion of novel plasmids.

## Plasmid establishment within populations

After acquisition by a recipient, attention turns to whether the plasmid can become established within the population. Successful establishment within populations—i.e. plasmid ‘stability’—is governed by the fitness effects of plasmids on their new hosts, alongside conjugation rate and loss through segregation.

Plasmid fitness costs—negative effects arising as a result of plasmid carriage—predominantly emerge through genetic conflict within the host, such as activation of the SOS response, in addition to translational demand and costs of conjugation [4,6,45]. Genes involved in these host-specific conflicts are still being uncovered, and can include supposedly beneficial accessory genes: for example, resistance gene *bla*_OXA-48_ carbapenemase, was mainly responsible for fitness costs across 13 multidrug Enterobacteriaceae carrying pOXA-48 [46]. Where plasmid-associated fitness costs are high, purifying selection will favour plasmid-free competitors, hindering the plasmids’s invasion. However, net plasmid carriage cost is not fixed. Fitness effects depend on the environment [45], and selection for plasmid-encoded accessory genes can dwarf fitness costs, driving the establishment of plasmids within new populations through positive selection for plasmid-carrying hosts. The long-term impact of positive selection will vary depending on the manner of the plasmid-encoded trait, with ‘private’ traits being better able to sweep to fixation, while ‘public goods’ traits, which benefit surrounding individuals, preventing fixation of the plasmid in the population [47]. Even without direct selection for plasmid-encoded traits, plasmid fitness effects can vary widely across related strains, sometimes even offering fitness benefits without specific selection for plasmid-encoded traits [48]. Coinfection with other plasmids can also have epistatic effects that reduce the cost of carriage, promoting establishment of a compatible plasmid within a plasmid-carrying host [49,50]. Fitness epistasis can interact with environmental selection, as co-existing plasmids can form colocalising clusters of plasmids with compatible traits, each benefiting from the accessory functions of other plasmids [51] Compensatory evolution—mutations that alleviate or mask fitness costs—can also stabilise plasmids long term, even after transient selection [2–4,37,52,53]. Plasmids can also benefit from other adaptations occurring to a host, for example, host mutations to adapt to changing or novel environments can reduce plasmid fitness cost (i.e. synergistic pleiotropy), allowing the plasmid to piggyback on host niche adaptation [3,54].

Conjugation imposes fitness costs, creating an inherent tradeoff between plasmid vertical transmission (i.e. to daughter cells) and horizontal transmission (i.e. to neighbouring cells). Most plasmids seem to operate below a ‘conjugation efficiency threshold’ [18], regulating expression of conjugative machinery to mitigate the cost of conjugation. Indeed, selection for reducing plasmid costs can drive loss of conjugation [55] Where plasmids become non-transmissible, this can lead towards an evolutionary dead end [6]. However, non-conjugative plasmids can hitchhike with coinfecting plasmids [6,56], and even non-mobilisable plasmids may be able to regain mobility by acquiring genes that promote transduction by phages [7,57]. In contrast, some plasmids can act as infectious elements, invading populations through high conjugation rates, resulting in dynamics resembling those of host–parasite systems [18]. Favourable conditions (high densities of recipient cells, population mixing) can sustain infectious plasmids in populations, at least in the laboratory [53,58–60].

## Plasmid establishment in the community

At the community level, plasmid spread represents both an upscaling of host and population level factors, and the introduction of multipartite interactions, including community diversity, competition, stability and spatiality.

**Figure 2:**
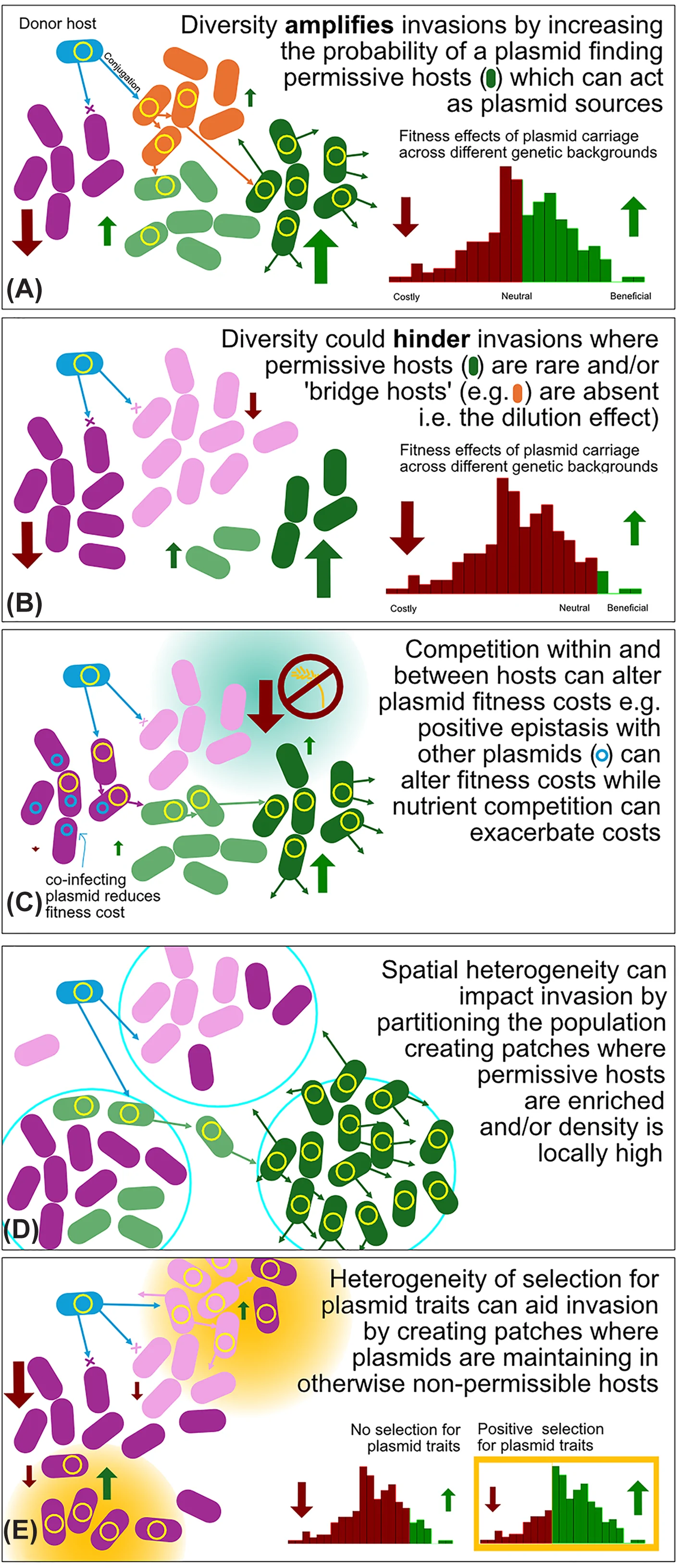
The impacts of the community on plasmid invasions Oblong shapes represent bacteria with connecting arrows representing conjugation. Histograms show hypothetical distributions of plasmid fitness effects with colour denoting positive (green) and negative (pink) effects. (**A**) Diverse communities harbour a wide distribution of fitness effects for a given plasmid, increasing the probability of plasmids finding permissive hosts (green bacteria). (**B**) Diversity may limit invasion where non-permissive hosts (pink bacteria) are common and block transmission even where permissive hosts are present. (**C**) Interactions between plasmids within hosts or between hosts for resources can alter fitness effects. (**D**) Spatial structure creates locally variable patches that, by chance, provide favourable conditions for plasmid spread e.g. higher density or enrichment of permissive hosts. (**E**) Diversity in selection for plasmid-encoded traits can enhance spread within areas of local plasmid benefit.

From a plasmid’s perspective, a diverse community is a collection of distinct plasmid–host interactions [48,61,62]: a single plasmid will encounter as many sets of parameters (such as fitness impact, conjugation rate etc.) as it has potential hosts, in some cases involving positive interactions amid distributions that are otherwise predominantly negative [48,63]. Community diversity, therefore, increases *variance* in fitness effects (Figure 2A) that can be more important than average values, allowing costly conjugative plasmids to persist within permissive hosts, which act as a reservoir for plasmid persistence at the community level [48,60]. This ‘source-sink’ dynamic has been demonstrated experimentally [58], and high-throughput sequencing approaches have shown that plasmids persist in complex communities through rare instances of positive fitness across diverse phylotypes [63].

The extent to which plasmids can ‘access’ hosts within a given community depends on host range, which varies from narrow host range plasmids like the Enterobacteriaceae-specific ColE1 plasmids [64], to the broad host range (BHR) plasmids like IncP, which can infect representatives of nearly all Gram-negative and some Gram-positive bacteria [5]. BHR plasmids are relatively common [65,66] and could have an advantage in a complex community, by being able to sample a wider diversity of hosts. However, evolutionary theory predicts that narrow host range plasmids may compensate by having a lower average cost and/or higher average benefit to their hosts, or be more successful in overcoming specific host defences, analogous to the generalist-specialist trade-off common among parasites [67,68].

Community diversity and host range will potentially interact in subtler ways too, by determining the connectedness of plasmid hosts within a community. Where **permissive hosts** are rare, transmission can be impeded by a ‘dilution effect’ [69], as too many individuals resistant to plasmid acquisition or maintenance will block the spread of a plasmid into a permissive sub-population [70] (Figure 2B). Lack of population mixing can also constrain plasmid transfer [71], though ecological factors that promote local coexistence, such as cross-feeding, can alleviate this effect [72]. Connectedness may also be influenced by the relatedness of potential hosts in the community, as plasmid transfer is more efficient between related bacteria [26]. Taxonomically intermediate hosts could increase the flow of plasmids between distantly related individuals, analogous to ‘bridge hosts’ in disease ecology, which facilitate pathogen spillover into new species [5,73,74].

Competition adds a further layer of complexity (Figure 2C). Plasmids compete within-cells for resources and plasmid–host combinations compete against other hosts and their resident plasmids [41]. Interspecies competition affects plasmid cost, with plasmids imposing a greater cost to a focal strain when grown in a community, compared to monoculture, ultimately driving greater plasmid loss [75]. However, under conditions where plasmids carry beneficial traits, plasmids might give their hosts an edge over competitors [62,63]. Competition can also be modulated by plasmid transmission: where donors have adapted to plasmid carriage, conjugation might impose fitness costs on a naive competitor, effectively ‘weaponising’ the plasmid in favour of the donor strain [76].

As the accessory genes carried by plasmids typically confer context-dependent fitness effects, the prevailing environmental conditions can dramatically alter species interactions within communities. Positive selection for plasmid-encoded traits can increase connectivity between plasmids and hosts within a multispecies network by increasing the fraction of the community in which the plasmid can be maintained [77,78]. However, the nature of the trait can modulate the outcome: ‘public goods’ traits, for example, can benefit plasmid-free neighbours [47], potentially allowing less accommodating community members to benefit without experiencing the burden of plasmid carriage. Traits for which selection is transient or fluctuating may also experience different dynamics to those under more consistent selection, with the former shown to favour invasion-through-transfer and the latter invasion-through-carriage [79]. Meanwhile, selection for protective traits, which act by killing individuals that lack them, could drive different dynamics than selection for traits that enhance growth. Analysis of plasmid accessory gene content across diverse microbial communities revealed a vast diversity of functions that differ across environments, with animal- and human-associated plasmids carrying more resistance, defence and repair functions, and plasmids from environmental communities being enriched for metabolic and growth related traits [80]. Thus, plasmid invasions may be governed by different mechanisms across different selective environments.

Finally, spatial heterogeneity promotes plasmid persistence through several intersecting mechanisms, though their interactions can produce apparently contradictory outcomes. High diversity at the scale of, for instance, a plant root or a throat swab, actually represents an aggregation of microhabitats each containing lower (alpha) diversity, but varying between one another (high beta dispersion). This distinction helps reconcile the observation that plasmid persistence can benefit from community diversity in some cases [48,58,60] but be limited by it in others [70,81]. Low diversity within localised patches increases the probability that permissive hosts are enriched in some by chance, enabling persistence in metapopulations where the plasmid would be lost from a homogeneous community (Figure 2D) [82]. Even within single-species populations, heterogeneity in host distribution can enhance transmission by increasing the cell density, and thus potential for transmission, in local patches (Figure 2D) [20] a pattern especially pronounced in high-structure environments such as biofilms and cell aggregates [20]. Spatial heterogeneity in plasmid fitness effects—such as uneven antibiotic distribution—can sustain plasmids in populations by creating areas of locally high fitness (Figure 2E) [83], though its consequences in mixed communities remain unexamined. Together, these factors generate a complex mosaic of selective pressures and fitness effects across habitats such as soil or the human gut, creating local patches of plasmid persistence. Whether these patches act as sources for broader community-wide dissemination or instead produce a silo effect—where permissive patches are rare and transfer between them limited—likely mirrors the diversity dynamics described above, and remains an important open question.

## Potential applications of plasmid invasions

As natural ecosystem engineers, plasmids offer the potential to transform our approach to manipulating microbial community function via metagenomic engineering. Conventional bacterial-level approaches, i.e. probiotics or inoculants, face a fundamental ecological problem: the introduced strain must establish within an existing community. This is especially challenging when inoculant strains are invading occupied niches [84] and competing against locally adapted communities. Probiotic strains are primarily chosen based on performance in simple systems for their desired function and not for their competitive fitness. And regardless, it is clearly a practical impossibility for a product to be tailored to the specific biotic and abiotic environment unique to each application. Thus, probiotic/inoculant approaches frequently fail to establish functional traits [85–87].

Plasmid-based approaches—termed ‘metagenomic engineering’, ‘plasmid-mediated bioaugmentation’ or ‘genetic bioaugmentation’—decouple the fate of the functional trait from the fate of the inoculating strain. Introducing a functional trait on a plasmid allows for the plasmid, and thus the trait, to invade the locally adapted community even when lost from the original host species [75].

This idea is far from novel. Plasmid-mediated bioaugmentation of catabolic functions has long been proposed as an approach for treating organic contaminants in wastewater and soils [11,88,89]. Many plasmids carry pathways for degradation of xenobiotic compounds, such as herbicides or chlorinated aromatics, and so can be introduced to polluted environments where needed. The success of this plasmid-mediated bioaugmentation has been demonstrated numerous times in closed systems, with a common feature often being the loss of the donor/inoculant bacteria over time, and the plasmid maintained within transconjugant lineages [90–92]. More recent applications have expanded to degradation of plastic pollution [93], or using CRISPR–Cas to target unwanted functions such as antibiotic resistance in microbial communities [94].

The design of bioaugmentation strategies is effectively a question of community invasion. As described above, successful outcomes from an engineering perspective could look very different at the ecological level: for example, community function may be achieved by the plasmid becoming stably established in a single abundant taxon and expressing its function at a high level, or the persistence of the plasmid in many rarer taxa, perhaps with lower functionality, maintained through high rates of HGT. It may even involve the plasmid backbone becoming lost, with retention of the trait via recombination onto local replicons. Thus, the design choices needed in developing such vectors for ecosystem change require careful consideration.

### Host range

The availability of potential hosts is critical for success, as hosts must be sufficiently accessible to allow transmission between permissive hosts or environments. Intuitively, BHR plasmids should be desirable as they can sample from a wider range of hosts, potentially increasing the probability of finding a ‘superhost’, increasing the redundancy in host choice and reducing the impact of the dilution effect. Plasmids that are specific to the most abundant clades in an ecosystem are also attractive, as, where diversity is largely skewed to specific taxa, this may in fact result in a higher potential host pool. Reliance on a narrow host pool, however, can increase the risks of failure resulting from the plasmid being unable to establish in target taxa, perhaps owing to the presence of defence systems [44]. Applications that require plasmids to establish within specific target taxa (such as reservoirs of AMR) are more susceptible to such risks compared with target-agnostic interventions that aim to establish a novel function in a community more generally.

### Fitness and function

Host range must also be considered beyond the capacity for a plasmid to infect a strain. Clearly, the ability of the trait to function across different recipients is a key factor, but variance in plasmid cost, which determines persistence within a population, is also important. In diverse communities, it is expected that plasmids will impose inhibitory costs on some hosts, or traits might be repressed or otherwise not function; however, as we have discussed, the distribution and magnitude of these effects across the host range are critical.

### Designer plasmids

Many strategies utilise the natural functionality of conjugative plasmids, either introducing plasmids that naturally carry traits of interest or selecting plasmids from representative environments with ideal traits to introduce novel traits onto, such as plastic degradation. Potentially, there is scope to ‘engineer in’ features that increase the invasion success of a plasmid. An elegant approach is to offer bacterial hosts a fitness ‘incentive’ to plasmid acquisition. For example, a recent study engineering AMR-targeting plasmids introduced an unrelated, metabolic function that is beneficial in the target environment, the *fos* operon. Inclusion of *fos* resulted in the maintenance of the plasmid, allowing for effective reduction of target AMR prevalence [95]. However, given the plasticity of plasmids, the potential for unforeseen consequences in complex microbial ecosystems, and the risks posed by microbial evolution, strategies based on recombining novel plasmid vectors for spreading genes in natural microbial communities should be approached with caution.

## Conclusion

The factors governing plasmid invasion into microbial communities are vastly more complex than those operating in single bacterium–plasmid systems, requiring an understanding of the properties of and variance between the many potential plasmid–host combinations, and situating these dynamics in a context of interactions at multiple scales and across heterogeneous landscapes. Scaling our understanding of plasmid invasion from the individual host to the community therefore represents one of the central challenges in microbial ecology that will require innovative approaches in synthetic biology, experimental evolution, finescale analysis of natural communities, and modelling to meet. This effort is certainly worthwhile. The ability to predict plasmid invasions and rationally design plasmid vectors for community-level invasion would transform our capacity to engineer microbiomes with applications in health, agriculture, and environmental management. Plasmids have been engineering microbial communities for billions of years; understanding and harnessing that process is one of the most promising frontiers in both fundamental and applied microbiology.

## Summary

- Many conjugative plasmids can invade multiple, perhaps many, populations within microbial communities. Thus, as agents of HGT, plasmid invasions can alter functionality across microbiomes
- Community invasions can be understood as an upscaling of dynamics with individual hosts. Variation in these dynamics across communities can either facilitate or hinder plasmid spread.
- Features of communities, such as spatial structure and competitive interactions between and within host populations, also alter plasmid invasion dynamics.
- Ultimately, being able to understand and predict plasmid invasion can help inform interventions to alter microbiome function, enabling us to tackle environmental challenges such as pollution, or target unfavourable microbial traits such as drug resistance.

## Open Access

Open access for this article was enabled by the participation of the University of Sheffield in an all-inclusive Read & Publish agreement with Portland Press and the Biochemical Society under an agreement with JISC.

## Abbreviations

AMR: antimicrobial resistance
BHR: broad host range
HGT: horizontal gene transfer
